# Associations of perceived neighborhood environment and physical activity with metabolic syndrome among Mexican–Americans adults: a cross sectional examination

**DOI:** 10.1186/s13104-020-05143-w

**Published:** 2020-06-26

**Authors:** Rodney P. Joseph, Sonia Vega-López

**Affiliations:** 1 Center for Health Promotion and Disease Prevention, Edson College of Nursing and Health Innovation, Arizona State University, 500 N 3rd Street, Phoenix, AZ 85004 USA; 2 College of Health Solutions and Southwest Interdisciplinary Research Center, Arizona State University, 500 N 3rd Street, Phoenix, AZ 85004 USA

**Keywords:** Built environment, Hispanic, Minority health, Exercise, Metabolic syndrome

## Abstract

**Objective:**

This secondary data analysis examined associations among perceived neighborhood environmental factors, physical activity (PA), and the presence of metabolic syndrome (MS) in Mexican–American (MA) adults. Seventy-five MA adults (mean age of 37.9 ± 9.3 years) provided anthropometric, biomarker, and survey data. The Neighborhood Scales Questionnaire evaluated six perceived neighborhood factors: walking environment, aesthetic quality, safety, violence, social cohesion, and activities with neighbors. The Rapid Assessment of PA questionnaire assessed PA. MS was determined according to ATP III criteria.

**Results:**

PA was significantly associated with MS (OR = .338, CI .204–.738). Neighborhood factors of safety (B = .255, p = .024), walking environment (B = .384, p = .001), and social cohesion (B = .230, p = .043) were positively associated with PA. No other neighborhood factors were significantly related to PA. Analyses examining whether neighborhood factors moderated the relationship between PA and MS were not significant.

## Introduction

Metabolic syndrome affects approximately 35% of Mexican–Americans [1] and is associated with increased risk for diabetes, cardiovascular disease, and premature death [2, 3]. Performing regular aerobic moderate-to-vigorous physical activity (PA) is an independent risk factor to prevent [4] and manage the collection of metabolic abnormalities implicated in metabolic syndrome [4–7]. Despite this benefit, PA levels of many Mexican–Americans remain at sub-optimal levels. This was recently highlighted with accelerometer-measured PA data from the Hispanic Community Health Study/Study of Latinos that showed approximately 42% of Mexican–Americans achieve national guidelines for aerobic activity (i.e., ≥ 150 min of moderate-intensity PA) [8]. However, this percentage was reduced to 18.7% when considering only PA performed in 10-min bouts. The low PA levels and high prevalence of metabolic syndrome among Mexican Americans highlight the need of a better understanding of the determinants of PA engagement among this high-risk population.

Extant research shows that physical and social environments influence human behavior. This is particularly the case for PA, where previous studies have demonstrated a consistent relationship between environmental factors and PA engagement [9–14]. Residential neighborhood characteristics, in particular, may influence PA behaviors given most individuals spend a substantial amount of time at home and in surrounding areas. Thus, providing the opportunity to support or discourage various types of PA, including leisure-time (i.e., neighborhood walking for exercise) and transportation-related (i.e. walking to work, taking children to school, grocery shopping) [13–15]. Although neighborhood factors have been shown to influence PA behaviors, few studies have examined whether such factors moderate the relationship between PA and metabolic disease risk [16]. This represents a gap in the literature given: (a) the potential impact neighborhood environmental factors have on PA, and (b) the fact that PA is a modifiable risk factor for developing metabolic abnormalities associated with metabolic syndrome. The objective of this secondary data analysis was to explore the associations among neighborhood environment factors (i.e., walking environment, aesthetic quality, safety, violence, availability of healthy foods, social cohesion, and activities with neighbors), PA, and the presence of metabolic syndrome among Mexican American adults. Data were obtained from a cross-sectional study [17] examining cardiometabolic risk factors and lifestyle habits among a convenience sample 75 community-dwelling Mexican–American adults. Specifically, we examined: (a) associations between PA and the presence of metabolic syndrome, (b) associations between neighborhood environmental factors and PA, and (c) whether neighborhood environmental factors moderated the relationship between PA and the presence of metabolic syndrome.

## Main text

### Methods

#### Participants

Inclusion criteria for the cross-sectional study (n = 75) [17] from which data were obtained included: (a) self-identifying as Mexican–American, (b) residing in the Phoenix metropolitan area for at least 12 months, and (c) being aged 21 to 60 years. Potential participants were excluded if they: (a) had a known chronic condition (i.e., diabetes, cardiovascular disease, cancer, hepatitis), (b) self-reported following a specific diet regiment (i.e., low carbohydrate, vegetarian), (c) were unable to walk for exercise, (d) were pregnant or breastfeeding, and (e) self-reported current participation in another research study where dietary and/or PA habits were assessed or manipulated.

#### Measures

*Demographics* Demographic characteristics of age and gender were collected using a questionnaire developed for the study.

*Perceived Neighborhood Environment* Perceived social and physical neighborhood characteristics were collected using the Neighborhood Scales Questionnaire [18]. This questionnaire is comprised of 7 scales assessing the following neighborhood characteristics: walking environment (7 items), aesthetic quality (5 items), safety (3 items), violence (4 items), availability of healthy foods (3 items), social cohesion (4 items), and activities with neighbors (4 items). Participants responded to survey items using a 1 to 5 scale (i.e., 1 = never, 2 = rarely, 3 = sometimes, 4 = most of the time, and 5 = always). Scales were scored individually by taking an average of scale items. Higher scores on these scales were associated with more favorable neighborhood characteristics, with the exception of the violence scale, where a higher score indicated greater levels of perceived neighborhood violence. The Neighborhood Scales Questionnaire has established test–retest reliability (i.e., ICCs ranging from .60 to 88) [18]. Internal consistency estimates (i.e., Cronbach alpha coefficients) for the scales ranged from .72 to .93 in the current study, which are comparable to previous research [18, 19].

*Physical Activity* The Rapid Physical Activity Questionnaire (RAPA) [20] assessed self-reported PA. This 9-item measures assesses aerobic (n = 7 items), muscle strengthening (n = 1 item), and stretching activities (n = 1 item). For the current study, only aerobic activity was included as the PA outcome, as this type of PA has shown to be most associated with cardiometabolic disease risk reduction [21–23]. The RAPA was scored by categorizing participants into one of five activity levels: 1 = sedentary (i.e., rarely or never does any PA), 2 = underactive (i.e., performs some light or moderate-intensity PA, but not every week), 3 = underactive regular/light (i.e., performs some light PA every week), 4 = underactive regular (i.e., engages in some moderate-intensity and vigorous-intensity PA, but less than 150 min/week of moderate-intensity PA or 60 min/week of vigorous-intensity PA), and 5 = active (i.e., engages in ≥ 150 min/week of moderate-intensity PA or ≥ 60 min/week of vigorous-intensity PA). The RAPA was previously validated against accelerometer-measured PA among Mexican Americans and demonstrated adequate test–retest reliability in the same sample (ICC.65, p = .002) [24].

*Metabolic Syndrome* National Cholesterol Education Program Adult Treatment Panel III (NCEP ATP III) [25] criteria were used to identify the presence of MS based on the presence of three or more of the following risk factors: 1) increased waist circumference (i.e., > 102 cm for men or > 88 cm for women); 2) hypertension (≥ 130/≥ 85 mmHg); 3) elevated triglycerides (i.e., ≥ 150 mg/dl); 4) low HDL cholesterol (i.e., < 40 mg/dl in men, < 50 mg/dl in women); and 5) impaired fasting glucose (≥ 100 mg/dl).

#### Procedure

Participants attended a single study assessment after fasting for at least 8 h. After written informed consent was obtained, anthropometric assessments (waist circumference and blood pressure) and a venous blood draw for biomarker measures (fasting plasma glucose, and serum triglycerides and HDL cholesterol) were conducted. Questionnaire data on perceived neighborhood environment and PA were collected via a bilingual interview in the participants preferred language (Spanish or English).

#### Statistical analysis

Descriptive statistics were calculated to summarize demographic characteristics, survey outcomes, and the presence of metabolic syndrome. Outcome analysis was performed in three phases using logistic and linear regression modelling techniques. All models controlled for covariates of age and sex, as previous research shows these factors are associated with PA levels (i.e., PA decreases with age; men engage in higher PA levels than women across the lifespan) [8] and the presence of metabolic syndrome (i.e., prevalence of metabolic syndrome increases with age, with men having a higher prevalence of metabolic syndrome than women until the approximate age of 50, at which point women have a higher prevalence than men) [26].

In phase 1 of analysis, logistic regression examined the association between PA and the presence of metabolic syndrome (i.e., binary outcome of having vs. not having metabolic syndrome). In phase 2, a series of linear regression models examined associations between perceived neighborhood characteristics and PA. For these models, associations between each neighborhood scale (i.e., walking environment, aesthetic quality, safety, violence, availability of healthy foods, social cohesion, and activities with neighbors) and PA were examined separately due to collinearity among subscales (see correlation matrix uploaded as an Additional file 1). In phase 3, hierarchical logistic regression models examined: a) the independent effect of each neighborhood factor on the presence of metabolic syndrome, and b) whether neighborhood factors moderated the relationship between PA and the presence of metabolic syndrome. Initial models included each neighborhood scale as an independent predictor for metabolic syndrome. Next, PA was entered into the model to predict of metabolic syndrome followed by an interaction term of neighborhood scale X PA (e.g. walking environment X PA). A significant interaction effect would indicate a moderating effect of the neighborhood factor on the relationship between PA and the presence of metabolic syndrome. Similar to linear regression models used in phase 2, separate models for each of the perceived neighborhood scale were conducted due collinearity among neighborhood scales.

### Results

Participants (n = 75) had a mean age of 37.6 ± 9.3 years and the majority were female (n = 49; 65.3%). Twenty-nine percent (n = 22) were classified as having metabolic syndrome. Table 1 describes descriptive statistics for PA, and perceived environmental factors.

**Table 1:** Descriptive statistics for perceived environmental factors and physical activity measures

Variable	Mean (SD)	Range
Perceived neighborhood factors (score)		
Walking environment	3.6 (0.9)	1.57–5.0
Safety	3.7 (0.9)	1.3–5.0
Aesthetic quality	3.9 (0.7)	2.2–5.0
Violence	1.4 (0.5)	1.0–3.5
Availability of healthy foods	3.6 (1.2)	1.0–5.0
Social cohesion	3.3 (0.9)	1.0–5.0
Activities with neighbors	2.5 (1.0)	1.0–4.8
Physical activity (RAPA score)	4.2 (0.9)	2.0–5.0

#### Physical activity levels and the presence of metabolic syndrome

PA levels were significantly associated with the presence of metabolic syndrome (OR = .388, CI = .204–.738). Individuals with higher PA levels were less likely to be classified as having metabolic syndrome.

#### Neighborhood environment and physical activity levels

Table 2 shows the results of the series of regression models evaluating associations between perceived neighborhood characteristics and PA. Neighborhood factors of walking environment (R^2^ = .173, B = .385, p = .001), safety (R^2^ = .093, B = .255, p = .024), and social cohesion (R^2^ = .081, B = .230, p = .043) were significantly related to PA levels. Participants who reported their neighborhood environment to be more conducive to walking, more safe, and having high levels of social cohesion engaged in higher PA levels. Perceived neighborhood factors of aesthetic quality, violence, availability of healthy food, and activities with neighbors were not significantly associated with PA.

**Table 2:** Linear regression models examining associations between perceived neighborhood characteristics and physical activity

Variable	B	SE	β	*P*
Walking Environment	.385	.108	.384	*.001*
Safety	.255	.110	.262	*.024*
Aesthetic quality	.214	.146	.175	.146
Violence	−.311	.188	−.192	.102
Availability of healthy foods	.153	.087	.203	.083
Social cohesion	.230	.111	.235	*.043*
Activities with neighbors	.191	.109	.202	.083

All models are adjusted for age and sex

The* p* values were italicized to indicate statistical significance

B indicates unstandardized coefficient

β indicates standardized coefficient

SE indicates standard error

*p* indicates *p* value

#### Neighborhood environment, physical activity and the presence of metabolic syndrome

Logistic regression analyses examining the independent effect of neighborhood characteristics on the presence of metabolic syndrome are presented in Table 3. Results showed none of the 7 neighborhood factors were significantly associated with the presence of metabolic syndrome. Subsequent models examining moderating effects of neighborhood factors on the relationship between PA and the presence of metabolic syndrome also failed to show significant moderation effects; indicating that none of the seven neighborhood factors moderated the relationship between PA and the presence of metabolic syndrome. Outcomes of these models are not presented due the numerous models tested and the non-significant outcomes. However, results of these models are available by contacting the first author.

**Table 3:** Logistic regression models examining associations of perceived neighborhood characteristics and presence of metabolic syndrome

		95% CI	
	OR	Lower	Upper
Walking environment	.637	.345	1.175
Safety	.725	.399	1.317
Aesthetic quality	.715	.338	1.515
Violence	1.240	.456	3.375
Availability of healthy foods	.740	.471	1.164
Social cohesion	.723	.404	1.293
Activities with neighbors	.715	.338	1.515

All models are adjusted for age and sex

OR indicates odd ratio, CI indicates confidence interval

### Discussion

Results showed that among our sample of Mexican American adults: (a) higher PA levels were inversely related to the presence of metabolic syndrome, and (b) perceived neighborhood safety, walking environment, and social cohesion were positively associated with PA levels. However, neighborhood factors of safety, walking environment, and social cohesion did not moderate the relationships between PA and the presence of metabolic syndrome.

The finding that higher self-reported PA was associated with reduced risk for the presence of metabolic syndrome adds to the established body of research showing PA is an independent risk factor associated with development of cardiometabolic disease conditions (i.e., metabolic syndrome and cardiovascular disease) [2, 5, 6, 27]. Results also confirm previous research showing that walking environment and safety are important neighborhood factors associated with PA engagement among adult populations (including Latinos) [11, 12, 28–31]; nevertheless, not all studies have reported significant associations among these variables [32]. Lack of significant relationships among neighborhood factors of aesthetic quality, availability of healthy foods, violence, and activities with neighbors with PA were surprising given these factors intuitively appear to be related to PA and previous research suggest several of these factors as important determinants of PA [28, 32, 33]. Inconsistent findings across studies may be related to measurement, as previous research has used numerous methods to measure both neighborhood factors and PA [11].

An interesting outcome was that none of the perceived neighborhood factors examined moderated the relationship between PA and the presence of metabolic syndrome. This was particularly unexpected among the variables of neighborhood walking environment, safety, and social cohesion given they were significantly related to PA. Reasons for this may be related to our relatively small sample size, the limited number of participants (n = 22) classified as having metabolic syndrome, and the possibility of participants over self-reporting PA on the RAPA. Another potential explanation is that factors not included in the current study mediate and/or moderate relationships among perceived neighborhood factors, PA, and the presence of metabolic syndrome. For example, extant research has shown that psychosocial processes (i.e., behavioral control, self-regulation, self-efficacy) play an important role in PA engagement [34–36]. It may be that interactions among PA-related psychosocial process and neighborhood environmental factors influence the relationship between PA and metabolic syndrome. Similarly, socioeconomic status [16] and environmental factors not included in our study, including mixed land use [16, 37], have shown to have interactive effects on both PA and metabolic disease risk. Future research examining the complex relationship among neighborhood environment, psychosocial processes, PA and metabolic disease risk is warranted.

## Limitations

Limitations include the cross-sectional design, use of subjective PA measures (although the RAPA has been validated against accelerometers for accurate assessment of PA among Mexican Americans [24]), and use of a relatively small convenience sample of Mexican Americans residing in a single geographic area.

## Supplementary information

**Additional file 1:** Correlations (Spearman’s) among individual scales on the Neighborhood Scales Questionnaire.

## Data Availability

The datasets used and/or analyzed during the current study are available from the corresponding author on reasonable request.

## Abbreviations

PA: Physical activity
OR: Odds ratio
ICC: Interclass coefficients

## References

[1] Heiss G, Snyder ML, Teng Y, Schneiderman N, Llabre MM, Cowie C (2014). Prevalence of metabolic syndrome among Hispanics/Latinos of diverse background: the Hispanic Community Health Study/Study of Latinos. Diab Care.

[2] Ford ES (2005). Risks for all-cause mortality, cardiovascular disease, and diabetes associated with the metabolic syndrome: a summary of the evidence. Diab Care.

[3] Mottillo S, Filion KB, Genest J, Joseph L, Pilote L, Poirier P (2010). The metabolic syndrome and cardiovascular risk a systematic review and meta-analysis. J Am Coll Cardiol.

[4] Zhang D, Liu X, Liu Y, Sun X, Wang B, Ren Y (2017). Leisure-time physical activity and incident metabolic syndrome: a systematic review and dose-response meta-analysis of cohort studies. Metabolism..

[5] Lakka TA, Laaksonen DE (2007). Physical activity in prevention and treatment of the metabolic syndrome. Appl Physiol Nutr Metab.

[6] Montesi L, Moscatiello S, Malavolti M, Marzocchi R, Marchesini G (2013). Physical activity for the prevention and treatment of metabolic disorders. Intern Emerg Med.

[7] Ostman C, Smart NA, Morcos D, Duller A, Ridley W, Jewiss D (2017). The effect of exercise training on clinical outcomes in patients with the metabolic syndrome: a systematic review and meta-analysis. Cardiovasc Diabetol.

[8] Arredondo EM, Sotres-Alvarez D, Stoutenberg M, Davis SM, Crespo NC, Carnethon MR (2016). Physical activity levels in Latino/Hispanic adults: results from the Hispanic Community Health Study/Study of Latinos. Am J Prev Med.

[9] Schulz A, Mentz G, Johnson-Lawrence V, Israel BA, Max P, Zenk SN (2013). Independent and joint associations between multiple measures of the built and social environment and physical activity in a multi-ethnic urban community. J Urban Health.

[10] Zapata-Diomedi B, Veerman JL (2016). The association between built environment features and physical activity in the Australian context: a synthesis of the literature. BMC Public Health.

[11] Barnett DW, Barnett A, Nathan A, Van Cauwenberg J, Cerin E (2017). Built environmental correlates of older adults’ total physical activity and walking: a systematic review and meta-analysis. Int J Behav Nutr Phys Act.

[12] Smith M, Hosking J, Woodward A, Witten K, MacMillan A, Field A (2017). Systematic literature review of built environment effects on physical activity and active transport - an update and new findings on health equity. Int J Behav Nutr Phys Act.

[13] McCormack GR, Shiell A (2011). In search of causality: a systematic review of the relationship between the built environment and physical activity among adults. Int J Behav Nutr Phys Act.

[14] Saelens BE, Handy SL (2008). Built environment correlates of walking: a review. Med Sci Sports Exerc.

[15] Lee C, Moudon AV (2004). Physical activity and environment research in the health field: implications for urban and transportation planning practice and research. J Plan Lit.

[16] Rutt CD, Coleman KJ (2005). Examining the relationships among built environment, physical activity, and body mass index in El Paso. TX. Prev Med.

[17] Dellaserra CL, Crespo NC, Todd M, Huberty J, Vega-Lopez S (2018). Perceived environmental barriers and behavioral factors as possible mediators between acculturation and leisure-time physical activity among Mexican American adults. J Phys Act Health.

[18] Mujahid MS, Diez Roux AV, Morenoff JD, Raghunathan T (2007). Assessing the measurement properties of neighborhood scales: from psychometrics to ecometrics. Am J Epidemiol.

[19] Keller C, Ainsworth B, Records K, Todd M, Belyea M, Vega-Lopez S (2014). A comparison of a social support physical activity intervention in weight management among post-partum Latinas. BMC Public Health.

[20] Topolski TD, LoGerfo J, Patrick DL, Williams B, Walwick J, Patrick MB (2006). The Rapid Assessment of Physical Activity (RAPA) among older adults. Prev Chronic Dis.

[21] Chudyk A, Petrella RJ (2011). Effects of exercise on cardiovascular risk factors in type 2 diabetes. Diab Care.

[22] Ismail I, Keating SE, Baker MK, Johnson NA (2011). A systematic review and meta-analysis of the effect of aerobic vs. resistance exercise training on visceral fat. Obes Rev.

[23] Brook RD, Appel LJ, Rubenfire M, Ogedegbe G, Bisognano JD, Elliott WJ (2013). Beyond medications and diet: alternative approaches to lowering blood pressure: a scientific statement from the American Heart Association. Hypertension.

[24] Vega-Lopez S, Chavez A, Farr KJ, Ainsworth BE (2014). Validity and reliability of two brief physical activity questionnaires among Spanish-speaking individuals of Mexican descent. BMC Res Notes.

[25] Expert Panel on Detection, Evaluation, and Treatment of High Blood Cholesterol in Adults. Executive Summary of The Third Report of The National Cholesterol Education Program (NCEP) Expert Panel on Detection, Evaluation, And Treatment of High Blood Cholesterol In Adults (Adult Treatment Panel III). JAMA. 2001. 285(19):2486-97. 10.1001/jama.285.19.2486 11368702

[26] Pucci G, Alcidi R, Tap L, Battista F, Mattace-Raso F, Schillaci G (2017). Sex- and gender-related prevalence, cardiovascular risk and therapeutic approach in metabolic syndrome: a review of the literature. Pharmacol Res.

[27] Ozemek C, Laddu DR, Lavie CJ, Claeys H, Kaminsky LA, Ross R (2018). An update on the role of cardiorespiratory fitness, structured exercise and lifestyle physical activity in preventing cardiovascular disease and health risk. Prog Cardiovasc Dis..

[28] Silfee VJ, Rosal MC, Sreedhara M, Lora V, Lemon SC (2016). Neighborhood environment correlates of physical activity and sedentary behavior among Latino adults in Massachusetts. BMC Public Health.

[29] Van Holle V, Van Cauwenberg J, Van Dyck D, Deforche B, Van de Weghe N, De Bourdeaudhuij I (2014). Relationship between neighborhood walkability and older adults’ physical activity: results from the Belgian Environmental Physical Activity Study in Seniors (BEPAS Seniors). Int J Behav Nutr Phys Act.

[30] Pratt M, Yin S, Soler R, Njai R, Siegel PZ, Liao Y (2015). Does perceived neighborhood walkability and safety mediate the association between education and meeting physical activity guidelines?. Prev Chronic Dis.

[31] Murillo R, Reesor LM, Hernandez DC, Obasi EM (2019). Neighborhood walkability and aerobic physical activity among Latinos. Am J Health Behav.

[32] Perez LG, Slymen DJ, Sallis JF, Ayala GX, Elder JP, Arredondo EM (2017). Interactions between individual and perceived environmental factors on Latinas’ physical activity. J Public Health.

[33] Martinez SM, Ayala GX, Patrick K, Arredondo EM, Roesch S, Elder J (2012). Associated pathways between neighborhood environment, community resource factors, and leisure-time physical activity among Mexican-American adults in San Diego California. Am J Health Promot.

[34] Young MD, Plotnikoff RC, Collins CE, Callister R, Morgan PJ (2014). Social cognitive theory and physical activity: a systematic review and meta-analysis. Obes Rev.

[35] Michie S, Abraham C, Whittington C, McAteer J, Gupta S (2009). Effective techniques in healthy eating and physical activity interventions: a meta-regression. Health Psychol.

[36] Choi J, Lee M, Lee JK, Kang D, Choi JY (2017). Correlates associated with participation in physical activity among adults: a systematic review of reviews and update. BMC Public Health.

[37] Baldock K, Paquet C, Howard N, Coffee N, Hugo G, Taylor A (2012). Associations between resident perceptions of the local residential environment and metabolic syndrome. J Environ Public Health.

