# Supplementary material for: Associations of perceived neighborhood environment and physical activity with metabolic syndrome among Mexican–Americans adults: a cross sectional examination
Source: BMC Res Notes. 2020 Jun 26;13:306. doi: 10.1186/s13104-020-05143-w (PMC7320589; doi:10.1186/s13104-020-05143-w)
Supplement: Supplementary file 1 — Additional file 1: Correlations (Spearman’s) among individual scales on the Neighborhood Scales Questionnaire. [file 13104_2020_5143_MOESM1_ESM.docx]

**Additional File 1.** Correlations (Spearman’s) among individual scales on the Neighborhood Scales Questionnaire.

|  | **Walking Environment** | **Safety** | **Aesthetic Quality** | **Violence** | **Availability of Healthy Foods** | **Social Cohesion** | **Activities with Neighbors** |
| --- | --- | --- | --- | --- | --- | --- | --- |
| **Walking Environment** | 1 | .515** | .520** | -.524** | .427** | .492** | .280* |
| **Safety** | .515** | 1 | .650* | -.622** | .264* | .554* | . 197 |
| **Aesthetic Quality** | 520** | .650* | 1 | -.604* | .291* | .543* | .224 |
| **Violence** | -.524** | -.622** | -.604* | 1 | -.239* | -.391** | .001 |
| **Availability of Healthy Foods** | .427** | .264* | .291* | -.239* | 1 | .358** | .162 |
| **Social Cohesion** | .492** | .554* | .543* | -.391** | .358** | 1 | .480** |
| **Activities with Neighbors** | .280* | . 197 | .224 | .001 | .162 | .480** | 1 |
